# Preparation and Characterization of an Oyster Peptide–Zinc Complex and Its Antiproliferative Activity on HepG_2_ Cells

**DOI:** 10.3390/md21100542

**Published:** 2023-10-18

**Authors:** Bo Peng, Zhu Chen, Yejia Wang

**Affiliations:** 1 Institute for Environmental and Climate Research, Jinan University, Guangzhou 511443, China; 2 Guangdong Ocean Association, Guangzhou 510220, China; 3 Guangdong Center of Marine Development Planning Research, Guangzhou 510220, China; yingyiou99@163.com

**Keywords:** oyster, peptide–zinc complex, chelation, antiproliferation, HepG_2_ cells

## Abstract

It is evident that zinc supplementation is essential for maintaining good health and preventing disease. In this study, a novel oyster peptide–zinc complex with an average molecular weight of 500 Da was prepared from oyster meat and purified using ultrafiltration, ultrasound, a programmed cooling procedure, chelating, and dialysis. The optimal chelating process parameters obtained through a response surface methodology optimization design are a peptide/zinc ratio of 15, pH of 6.53, reaction time of 80 min, and peptide concentration of 0.06 g/mL. Then, the structure of a peptide–zinc complex (named COP2-Zn) was investigated using the UV and infrared spectrums. The results showed that the maximum absorption peak was redshifted from 224.5 nm to 228.3 nm and the main difference of the absorption peaks was 1396.4 cm^−1^. The cytotoxicity and antiproliferative effects of COP2-Zn were evaluated. The results showed that COP2-Zn had a better antiproliferative effect than the unchelated peptide against HepG_2_ cells. A DNA flow cytometric analysis showed that COP2-Zn induced S-phase arrest in HepG_2_ cells in a dose-dependent manner. Additionally, the flow cytometer indicated that COP2-Zn significantly induced HepG_2_ cell apoptosis.

## 1. Introduction

Zinc is an important trace element in the human body and presents in all tissues and body fluids [1]. Zinc ions are a key component of about 300 enzymes [2] which account for a series of enzymatic reactions and metabolic activities [3], such as aerobic oxidation, cell signaling pathways, free radical scavenging and metabolism, and DNA synthesis and repair [4], as well as for the normal function of cells mediating innate immunity [5]. However, conditional zinc deficiencies due to an insufficient dietary intake or low utilization is still common in some developing countries [6,7].

Previous studies have found that inorganic zinc can change the flavor of food and cannot be effectively absorbed in the gastrointestinal tract [8]. High doses of zinc ions, which have stronger toxicity, can stimulate the gastrointestinal tract and are accumulated in the body [9]. Therefore, it is necessary to find a safe and effective zinc supplementation. Zinc–peptide complexes have been proven to enhance the bioavailability, absorption efficiency, and safety of minerals [10]. Peptides can be potentially produced from different food-derived proteins, such as sesame [11], wheat germ [12], rapeseed [13], and loach peptides [14]. There are many metal ions that can be used to prepare metal chelates, including iron [15], zinc [16], calcium [17], and copper [18]. Udechukwu and others [19] found that the zinc from zinc–peptide complexes was absorbed better than that from inorganic zinc salts. Peptide-metal complexes have become the research spot in recent years due to their functional variety.

Oyster (ostrea gigas thunberg), a member of the ostreidae family, is a good source of bioactive peptides [20]. Studies have shown that oyster peptides have a variety of physiological activities, including zinc-binding, antiproliferative, anti-oxidant, hypotensive, and antibacterial activities [21,22]. These oyster peptides are rich in amino acids with metal chelating ability, such as histidine, cysteine, arginine, and so on [23]. Nevertheless, the previous research on oyster peptide–zinc complexes has mainly focused on anti-oxidation and anti-aging activities. However, the antitumor activity has not been fully studied [24]. In addition, its related mechanisms remain unknown. Hepatocellular carcinomas are one of the most frequent tumors in the world, and it poses a significant global healthcare burden [25]. Reports have shown that hepatocellular carcinomas require many approaches to establish the best treatment as well as the timing of multiple therapies for better results [26]. Therefore, further study needs to be conducted on cell proliferation as a target in carcinogenesis. Thus, we analyzed the antiproliferative effect of oyster peptides and their related mechanisms.

The objective of this study is to prepare and identify a novel peptide–zinc complex, which was mainly prepared by alkaline protease hydrolysis and an ultrasound-assisted and programmed cooling chelating procedure. The preparation process was optimized by using response surface methodology. The structural information of the chelating peptide was determined. The amino acid composition was determined by an automatic amino acid analyzer. The chemical structure changes were determined by ultraviolet and infrared absorption spectroscopy. The antitumor activity was further studied.

## 2. Results and Discussion

### 2.1. Optimization of Zinc–Peptide–Zinc Complex Process

**Figure 1:**
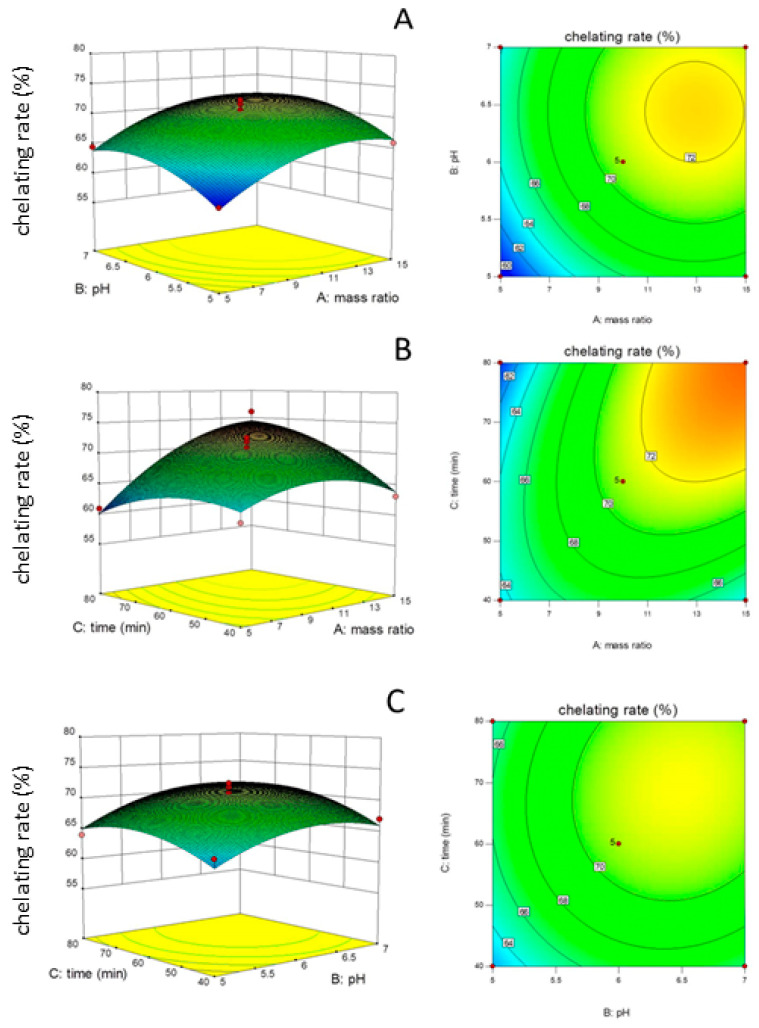
Three-dimensional diagram and contour map of effect of interaction between peptide–zinc ratios and pH values, peptide–zinc ratios and reaction times, and reaction times and pH values. (**A**) pH values and peptide–zinc ratios; (**B**) reaction times and peptide–zinc ratios; (**C**) reaction times and pH values.

In order to prepare the oyster peptide–zinc complex with higher chelation rates, the process parameters were optimized by using a response surface methodology, based on the results of single factor experiments (ultrasound time, temperature, and power; peptide/zinc ratio, pH value, and initial chelating temperature), which can be seen in Table 1 and Figure 1. Then, the peptide/zinc ratio, pH value, and reaction time were selected to optimize the zinc chelating process. According to the design of Design Expert 8.0.6, a total of 17 experiments were performed. As shown in Table 2, the model is significant. The lack of fit was larger than 0.05, which indicated that the quadratic polynomial response surface model had been successfully established. The regression equation can be obtained as follows:

$$\begin{array}{ll} Chelating\;rate\;(\%) & =-55.24+1.21A+32.67B+0.29C-7.16\times 10^{-3}AB+0.034\times AC\\ & +0.025\times BC-0.13\times A^{2}-2.65\;\times B^{2}-5.78\times 10^{-3}\times C^{2} \end{array}$$

It was found that the process of programmed cooling to prepare the oyster peptide–zinc complex can increase the chelation rate. The chelation reaction is an exothermic reaction, and the cooling is beneficial to the forward direction of the reaction. From the analysis of the significance level of each factor, the order of influence of each factor on the chelation rate is A > B > C, showing that the peptide–zinc ratio had the most significant effect on the chelation rate. Among all the square terms, A^2^, B^2^, and C^2^ had significant effects on the chelating rate (*p* < 0.05). In terms of interactions, only AB and AC had significant effects (*p* < 0.05). The optimal process conditions for oyster peptide chelating zinc were obtained: a peptide–zinc ratio of 15, pH of 6.53, and time of 80 min. The mean value of the results of three parallel experiments showed that the actual chelating rate was 74.13%, without a significant difference from the predicted values of the model, indicating that the chelating conditions of the oyster protein zinc chelate are feasible. Actually, many studies have been conducted on the optimization of polypeptide–Zn chelating. Li et al. (2019) made effective zinc-binding peptides from oyster-modified hydrolysates by adding exogenous glutamate, and the content of zinc reached 101.08 ± 3.10 mg/g [27]. Owing to the different sources of the polypeptides, the chelating rate and conditions also varied. Sun et al. (2021) obtained a novel peptide–Zn (AKP-Zn) chelate with conditions of pH 6.0 and 60 °C [28]. The zinc-binding peptides from mung bean protein hydrolysate have also been studied, and the maximal level of zinc chelating activity was 80.82 mg/g [29].

### 2.2. Analysis of Amino Acid Composition

As can be seen in Table 3, there are 18 kinds of amino acids including 9 kinds of essential amino acids in COP2-Zn, and the tryptophan had been destroyed and hadn’t been detected during the hydrolysis. The amino acids which have been reported to have a better zinc chelating ability were abundant in COP2-Zn, including aspartic acid (2.7%), glutamic acid (2.5%), glycine (1.7%), and proline (1.5%). The lysine (1.26%) content was higher than that of any other amino acid, which is similar to the results reported by Huang [30]. The amino acid composition is closely related with the chelating activities with metal ions. The contents of the above amino acids in the oyster peptides were relatively high, indicating that oyster peptide is a good source for peptide–zinc complexes.

### 2.3. Optimization of Zinc–Peptide–Zinc Complex Process

#### 2.3.1. Ultraviolet (UV) Spectroscopy Analysis

**Figure 2:**
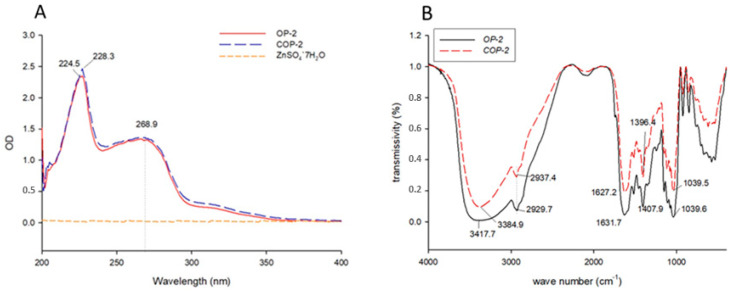
UV-Vis and infrared spectra of oyster peptide and zinc-chelating peptide complex. (**A**) UV spectra; (**B**) Infrared spectra.

Ultraviolet absorption is an effective method to evaluate the characteristic structures of substances. The change in the formation of peptides and peptide–metal complexes can be judged by the chemical shift and absorption intensity changes of a UV absorption spectrometer [31]. It can be seen from Figure 2A that the maximum ultraviolet absorption wavelength of the oyster peptide before and after chelation has changed, and the maximum absorption peak of the oyster peptide solution shifted from 224.5 nm to 228.4 nm. This may be due to interactions, involving the chirality of chromospheres and auxiliary pigments of C=O COOH -OH and -NH_2_, which is the main structure of peptides with a zinc-binding ability [32]. The maximum absorption wavelength shifted in the long wave direction and the absorption intensity increased. The experimental results of measuring the zinc sulfate solution showed no absorption in the ultraviolet region. The above results are similar to those of Yang’s reports [33], which indicated that new substances were produced after reaction with zinc ions.

#### 2.3.2. Fourier Transform Infrared Spectroscopy

Infrared absorption spectroscopy can be used to evaluate the chemical structure and composition of substances, even to quantitatively analyze some characteristic functional groups, such as O-H, N-H, C-O, etc. [34]. The FTIR spectra of the COP2-Zn and OP-2 are shown in Figure 2B. It can be seen from Figure 2B that before and after chelation, the amide N-H at 3419.6 cm^−1^ and at 1631.7 cm^−1^ had obvious characteristic absorption peaks in tensile vibration, which may be due to the chelation of N-H bending vibration, indicating the existence of an N-H structure in the oyster chelating peptide [34]. It can be seen that the peak that appeared at 1396.4 cm^−1^ became shallower and shifted to the higher frequency at 1407.9 cm^−1^ which was attributed to the stretch of the -COO- to combine with Zn^2+^ in forming COP2-Zn. The existence of tensile vibration at 1407.9 cm^−1^ indicated that the chelating peptide had a -COO structure. Before and after chelating, the main difference in the absorption peak was shown in the form of 11,396.4 cm^−1^. After chelating, the stretching vibration of the carboxyl group was blue-shifted and disappeared in situ, which was similar to the results of Udechukwu and others [35] and indicated that a chelating reaction between oyster peptide and zinc ions might be involved. In addition, the absorption peak of the chelating peptide was narrowed near 3439.6 cm^−1^, indicating that there was no exposed COOH in the chelating peptide. Apparently, the effect of OP-2 binding with Zn^2+^ on the spectra of OP-2 was owing to the formational changes of the COP2-Zn chelates. To sum up, it was possible that the principal binding sites of COP2-Zn were the carboxyl oxygen, amino nitrogen, hydroxyl oxygen, and oxygen of the peptides, which were involved in the formation of COP2-Zn.

### 2.4. Cytotoxicity

**Figure 3:**
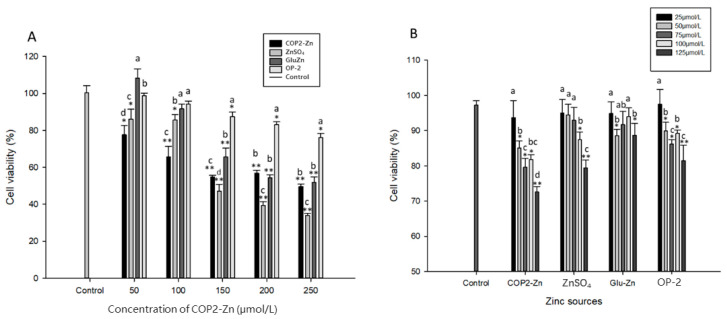
The cytotoxicity and antiproliferation activities of COP2-Zn, ZnSO_4_·7H_2_O, Glu-Zn and OP-2 against HepG_2_ cells. (**A**) Cytotoxicity; (**B**) antiproliferation activity. ^a–d^ Values with different letters are significantly different in each group (*p* < 0.05), * represents the significant difference compared with the control group (*: *p* < 0.05, **: *p* < 0.01).

Cytotoxicity is a cell killing event caused by a chemical substance (or drug) acting on a cell. Usually, the cytotoxic concentration of a chemical substance is evaluated by measuring cell viability [36]. Figure 3A shows the toxic concentrations of the peptide–zinc complex on HepG_2_ cells in the gastrointestinal tract followed by simulated digestion. It can be seen from Figure 3A that the effects of the concentrations of OP-2, COP2-Zn, zinc sulfate, and zinc gluconate on HepG_2_ cell growth are different. When the concentration was lower than 100 mol/L, the toxicology on HepG_2_ cells was detected to decrease. After simulating gastrointestinal digestion, the COP2-Zn could significantly inhibit the growth of HepG_2_ cells at a lower concentration (*p* < 0.05), and its antiproliferative activities slowly changed with increasing concentration. At the test concentration, the maximum cell viability was 43.7%. The antiproliferation activity of the zinc gluconate was lower than that of the polypeptide zinc and zinc sulfate at the different zinc concentrations.

### 2.5. Antiproliferation Activity

Different concentrations of zinc were treated with human liver cancer cells HepG_2_, and their antiproliferative activities were tested after 72 h. The results showed that at a certain zinc concentration, different kinds of zinc and different concentrations of zinc had antiproliferative activities on HepG_2_ cells in a dose-dependent manner, which was similar to the results of Liao [24]. The antiproliferative activities of zinc from different sources were as follows: COP2-Zn > ZnSO_4_ > zinc gluconate > OP-2, while the unchelated oyster peptide had a lower antiproliferative activity on HepG_2_ cells than that of the peptide–zinc complex. The peptide–Zn complex had the strongest antiproliferative activity (72.23%) compared to the other sources of zinc. As shown in Figure 3B, OP-2 had little antiproliferative impact on the HepG_2_ cells, meaning that the antitumor ability of COP2-Zn was considerably stronger than that of OP-2 and that the synthesis of OP-2 and Zn contributed significantly to the antiproliferative activity.

### 2.6. Effect of COP2-Zn on Intracellular Antioxidant Enzymes

**Figure 4:**
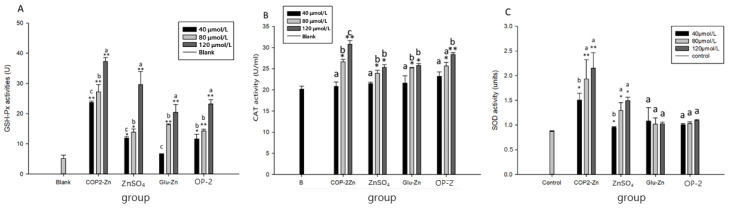
Effects on antioxidase activities of HepG_2_ cells treated with different concentrations of COP2-Zn. (**A**) Glutathionase activity; (**B**) catalase activity; (**C**) superoxide dismutase activity. ^a–c^ Values with different letters are significantly different in each group (*p* < 0.05), * represents the significant difference compared with the control group (*: *p* < 0.05, **: *p* < 0.01).

The ability of an antiproliferative active substance to enhance the cell antioxidant enzyme system is an important indicator for evaluating its ability to protect cells from oxidative damage. CAT, SOD, and GSH-PX are the key enzymes among cell antioxidant enzymes. Zinc metabolism homeostasis is found in many types of cancer, such as liver cancer, breast cancer, cervical cancer, and so on [20]. Zinc ions can inhibit tumor cell proliferation by inhibiting the expression of the wt-p53 protein, increasing SOD activity, reducing MDA content, inducing apoptosis, blocking cell division, and reducing angiogenesis [21]. As previously reported, the production of reactive oxygen species can cause disturbances in the antioxidant enzyme system, including for CAT, GSH-Px, SOD, and so on [37]. Previous work has shown that oyster-derived peptide–zinc complexes can regulate antioxidant activity. As shown in Figure 4, the activities of CAT, SOD, and GSH-Px in HepG_2_ cells pretreated with a peptide–zinc complex increased significantly in a dose-dependent manner. The activities of CAT, SOD, and GSH-Px in the peptide–zinc complex treatment group and the blank group had the highest values at a concentration of 120 mol/L. The peptide–zinc complex-treated group had the highest CAT activity in the HepG_2_ cells, compared with the blank group and the ZnSO_4_·7H_2_O- and Glu-Zn-treated groups. When treated with the peptide–zinc complex, the GSH-Px activities increased from 23.71 ± 0.46 U to 37.30 ± 1.31 U. When treated with the ZnSO_4_·7H_2_O, the GSH-Px activities increased from 11.89 ± 0.46 U to 29.6 ± 4.34 U. When treated with the Glu-Zn, the GSH-Px activities increased from 6.67 ± 0.07 U to 20.5 ± 2.55 U. All results indicated that the CAT, GSH-Px, and SOD activities were significantly increased by the peptide–zinc complex treatment in a dose-dependent manner. This proved that the COP2-Zn could promote antiproliferation by promoting the activity of antioxidant enzymes and clearing the intracellular reactive oxygen species.

### 2.7. Induction of Cell Apoptosis by COP2-Zn

**Figure 5:**
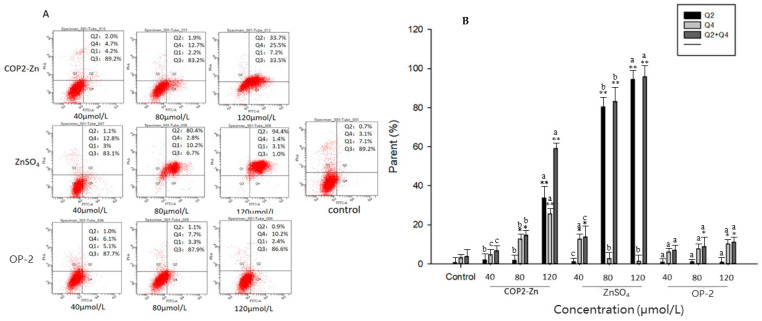
Effect of COP2-Zn on the apoptosis of HepG_2_ cells. (**A**) Cell apoptosis, (**B**) distribution of apoptotic cells. ^a–c^ values with different letters are significantly different in each group (*p* < 0.05), * represents the significant difference compared with the control group on each cell type (*: *p* < 0.05, **: *p* < 0.01).

As is known to all, cell apoptosis and necrosis are the two main forms of cell death, of which apoptosis plays a dominant role in these process as a form of programed cell death. The unique characteristics of apoptotic cells are cell volume contraction, chromatin aggregation, detachment, and phagocytosis from surrounding tissues, without an inflammatory response. Previous studies have shown that peptide–zinc complexes could induce HepG_2_ cell apoptosis and necrosis. To further verify whether peptide–zinc complexes can induce apoptosis or necrosis-mediated cell death, an Annexin V-FITC/PI reach assay was performed to detect COP2-Zn-treated cells. HepG_2_ cells were treated with COP2-Zn for 48 h, then stained with Annexin V-FTIC and PI, and detected by flow cytometry. In Figure 5A, the Q1, Q2, Q3, and Q4 quadrants represent necrotic cells, early apoptotic cells, normal cells, and late apoptotic cells, respectively. As shown in Figure 5B, as the concentrations of COP2-Zn, OP-2, and ZnSO_4_·7H_2_O increased, the apoptosis rate increased rapidly. When the concentration of these three fractions reached 120 μmol/L, the proportion of apoptotic cells increased from 3.8% to 59.2%, 95.8%, and 11.1%, respectively. When the concentration of COP2-Zn was less than 80 μmol/L, most apoptotic cells were concentrated in the Q2 region. As the concentration of COP2-Zn continued to increase, the cells tended to concentrate in the Q4 region, which indicated that a low concentration of COP2-Zn induced early apoptosis and a high concentration induced late apoptosis and necrosis. These results are similar to those reported in the study by Liao and others [24]. However, the apoptosis of ZnSO_4_·7H_2_O-treated cells was mainly concentrated in the Q2 region, suggesting that it induced early apoptosis. The apoptosis rate of the experiment groups was significantly higher than that in the control group in a dose-dependent manner, suggesting that COP2-Zn and ZnSO_4_·7H_2_O might promote apoptosis. Therefore, in order to induce the apoptosis and necrosis of HepG_2_ cells, the effective concentration of COP2-Zn (Zn^2+^) for cancer treatment and prevention should be less than 120 μmol/L. The ZnSO_4_·7H_2_O-treated cells seem to have similar activities in inducing the apoptosis of HepG_2_ cells compared with the COP2-Zn group. Research has discovered that zinc (Zn) has extensive anti-tumor effects in a variety of tumor cell lines [38,39]. Overdoes of zinc ions, on the other hand, may cause toxicity and adverse effects [24]. As a result, zinc dosage management and the combination of zinc with bioactive chemicals are critical for its clinical application. Studies have shown that oyster peptides have a variety of physiological activities [22]. Thus, we used OP-2 combined with zinc to conduct the work, which not only reduced the toxicology of zinc but improved the anti-cancer activities.

### 2.8. Effect of COP2-Zn on the Cell Cycle

**Figure 6:**
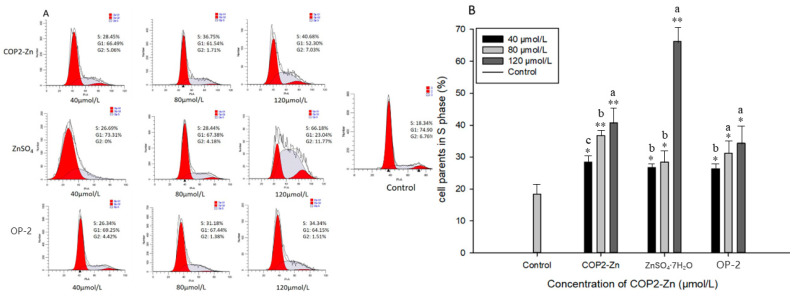
Effect of COP2-Zn on the apoptosis of HepG_2_ cells. (**A**) Cell apoptosis, (**B**) distribution of apoptotic cells. ^a–c^ values with different letters are significantly different in each group (*p* < 0.05), * represents the significant difference compared with the control group (*: *p* < 0.05, **: *p* < 0.01).

Changes in cell cycle distribution are also important indicators of apoptosis. The effects of COP2-Zn, ZnSO_4_·7H_2_O, and OP-2 on cell cycle distribution were determined by PI staining with flow cytometry. As shown in Figure 6, the cell cycle was significantly stagnated in the S phase in a dose-dependent manner after treatment with COP2-Zn, ZnSO_4_·7H_2_O, and OP-2. As the concentration of the COP2-Zn increased from 40 μmol/L, to 120 μmol/L, the density of the HepG_2_ cells in the S phase increased from 28.45% to 40.68%. As the concentration of ZnSO_4_·7H_2_O increased, the cell density increased from 28.44% to 66.18%. When the concentration of OP-2 increased, the cell density increased from 26.34% to 34.34%. Compared with the cell content of the blank group in the S phase of 18.34%, the proportion of cells in the treated sample increased significantly, which indicated that the COP2-Zn, ZnSO_4_·7H_2_O, and OP-2 blocked the cell proliferation in the S phase. This finding is consistent with the results of apoptosis in vivo, and also shows that ZnSO_4_·7H_2_O has a higher activity in blocking HepG_2_ cells than COP2-Zn and OP-2. In conclusion, the antiproliferative effects of COP2-Zn, ZnSO_4_·7H_2_O, and OP-2 mainly induced cell cycle arrest in the S phase. A similar result was found in the inhibition of ascitic tumors [40], suggesting that Zn complexes are a promising cancer agent for the future. Zhang et al. showed that thonningianin A triggered G0/G1 cell cycle arrest in HepG_2_ cells by altering cyclin D1 and CDK4 expression levels [41]. Therefore, more research on cyclin protein expression should be conducted.

## 3. Materials and Methods

### 3.1. Chemicals and Regents

Oysters were obtained from Guangzhou Huangsha seafood market (Guangzhou, China). Alcalase 2.4L (P4860, ≥2.4 U/g) was obtained from Novozymes Biotechnology Co., Ltd. (Copenhagen, Denmark). Zinc sulfate was purchased from Tianjin Damao Chemical Reagent Co., Ltd. (Tianjin, China). Lead reagent and methylene blue were obtained from Yuanye Biotechnology Co., Ltd. (Shanghai, China). Xylenol orange was purchased from Shanghai Jinsui Biotechnology Co. Ltd. (Shanghai, China). Phosphate-buffered saline (PBS, pH 7.4), penicillin, Dulbecco’s modified Eagle medium (DEME), and streptomycin were purchased from Gibco Life Technologies (Grand Island, NY, USA). Fetal bovine serum (FBS) was purchased from Hangzhou Sijiqing Bioengineering Materials Co. Ltd., (Hangzhou, China). The human hepatocellular carcinoma (HepG_2_) cell line was purchased from Southern Medical University. Catalase (CAT), glutathione peroxidase (GSH), superoxide dismutase (SOD), cell cycle, and apoptosis assay kits were purchased from Beyotime Biotechnology Co., Ltd. (Nanjing, China). All reagents used were of an analytical grade.

### 3.2. Extraction and Preparation of Oyster Peptides (Named OP-2)

The oyster meat was separated from the fresh oysters and washed. Then the internal organs were discarded, and the meat was stirred in a meat grinder (S2-A81, Jiuyang, Hangzhou, China). Finally, the minced meat was grinded and homogenized again with 3 times the volume of deionized water. Then the pH value was adjusted to 8.31, and 2.12% (*w*/*w*) Alcalase alkaline protease was added, then the oysters were immediately hydrolyzed in a water bath (SHA-CA, Xiuhua, Changzhou, China) at 55 °C for 5.19 h. At the end of the reaction, the polypeptide hydrolysate was heated and kept boiling for 15 min to stop the hydrolysis reaction. Then, the hydrolysate was cooled in an ice bath quickly, the pH value was adjusted to 7.0, the hydrolysate was centrifuged (Allegra X-15R, Beckman Coulter, Pasadena, CA, USA) at 4500 rpm for 15 min, and then the supernatant was taken out [30] The supernatant was filtered through a 5000 Da cellulose membrane (ultrafiltration system Millipore, Bedford, MA, USA) to obtain the peptide fractions of the oyster hydrolysate: >5K Da (named OP-1) and <5K Da (named OP-2). The obtained supernatant was concentrated by a reduced pressure rotary evaporator (Hei-VAP Value Digital, Heidoph, Germany) to 1/10 of its original volume at 60 °C. Based on the results of the single-factor optimization test, OP-2 was used as the optimized component for the subsequent tests and stored in a −40 °C refrigerator for further analysis.

### 3.3. Optimization in the Production of the Peptide–Zinc Complex (COP2-Zn)

Before the chelation reaction, the oyster polypeptide solution was subjected to ultrasonic (KQ-200KDV, Shumei, Tianjin, China) treatment with parameters: power 400 W, peptide concentration 0.1 g/mL, processing time 30 min, and temperature 40 °C. Based on the single-factor optimization experiments, Box–Behenken principle, and response surface design, the peptide/zinc ratios (5, 10, 15), pH values (5.0, 6.0, 7.0), and reaction times (40 min, 60 min, 80 min) were selected to optimize the zinc chelating process. And the chelating rate was used as an evaluation index. After adjusting the peptide solution concentration and pH value, it was mixed with zinc sulfate and fully preheated to a certain temperature. Next, the temperature was decreased at a gradient of 0.3 °C/min, and the temperature-controlled chelation reaction was performed. When the temperature dropped to 37 °C, the solution was incubated for 20 min in a water bath shaker (SHA-CA, Xiuhua Instrument Co., Ltd., Changzhou, China). The peptide–zinc complex was concentrated and further purified by dialysis (Spectra/Por Membranes, MW cutoff 500 Da, Spectrum Laboratory, Rancho Dominguez, CA, USA) and freeze-dried (ALPHA-1-2LD, Christ, Germany) to obtain the zinc–peptide–zinc complex (named COP2-Zn).

### 3.4. Determination of Zinc Chelating Capacity

The zinc chelating capacity was determined according to methods with slight modifications [13]. After the chelating reaction, 50 mL of the solution was poured into a conical flask, and 2 mL of a saturated ammonium fluoride solution, 0.5 mL of a 0.2 g/mL thiourea solution, 1 mL of a 0.04 g/mL vitamin C solution, 3 mL of a buffer solution, and 2 drops of xylenol orange were added in this order. The solution was mixed well and titrated with 0.002 M EDTA until the color changed from purple to yellow.

The obtained chelating peptide powder (0.05 g) was dissolved in distilled water and made into a 1 mg/mL solution for analysis of the Zn^2+^ content of COP2-Zn. Then the Zn^2+^ content was estimated by atomic absorption spectrophotometry (240 A, Agilent, Santa Clara, CA, USA).

$$Zinc\;chelating\;rate\;(\%)=\frac{amount\;of\;chelated\;{Zn}^{2+}}{total\;amount\;of\;{Zn}^{2+}}\times 100\%$$

### 3.5. Analysis of Amino Acid Composition

Amino acid composition was analyzed according to the method described by [42]. An Agilent 1100 Series HPLC (Palo Alto, CA, USA) with an automatic ALS liquid sampler, fluorescence detector (FLS), and diode array detector (DAD) was used. The samples were centrifuged at 4000 rpm for 10 min at 20 °C. A 5 mL sample and 100 μL of glutamic acid were then mixed with 100 μL of creatine as an internal standard. The mixture was filtered using a 0.45 μm OlimPeak pore filter (Teknokroma, Barcelona, Spain), a pre-column derivatization of diacetaldehyde phthalate (OPA reagent, Agilent), and a 9-methylfluoromethane Agilent FMOC reagent. The separation was performed at 40 °C, and 10 μL of the derivative sample was injected into a chromatography column (250 × 4.0 mm, inner diameter 5 μm).

### 3.6. UV–Visible and Fourier Transform Infrared (FTIR) Spectroscopy

To determine whether then zinc ion was chelated onto the peptide, UV spectroscopy measurements were performed according to the method described by Xie [13], with some modification. The COP2-Zn sample and a mixture of OP-2 and ZnSO_4_·7H_2_O were dissolved in a 0.1 M Tris-HCl (pH 6.0) solution. The solution was filtered through a 0.22 μm ultrafiltration membrane and scanned in the range of 200–400 nm with a UV spectrophotometer (E-201-c-9, Shanghai Ruosull Technology Co., Ltd., Shanghai, China). A Tris-HCl solution was used as a blank and the absorption spectrum of the zinc sulfate solution was scanned.

The prepared oyster polypeptide and peptide–zinc complex (both 1 mg) were mixed with 200 mg of KBr in an agate mortar, and sufficiently ground to a particle size of less than 0.25 μm. Then the mix was put into a tablet compression mold, and kept at 18 MPa for 3 to 5 min to obtain a transparent KBr tablet. Then, the tablet was shifted to the infrared test room, and scanned under a Fourier infrared spectrometer (Vector 22, Bruker, Mannheim, Germany) in the wave number range of 400–4000 cm^−1^ [43].

### 3.7. Cell Lines and Culture Conditions

Human HepG_2_ cells were obtained from the Animal Center Laboratory of Sun Yat-sen University (Guangzhou, Guangdong). HepG_2_ cells were cultured in 75 cm^2^ flasks in a DMEM growth medium containing 10% FBS (Gibco Life Technologies, Grand Island, NY, USA) and 1% penicillin/streptomycin antibiotic mixture. The cells were kept in a humidified incubator (Thermo Electron Co., Waltham, MA, USA) with an atmosphere with 95% air and 5% CO_2_ at 37 °C. The growth medium was changed three times a week, and passaged when the cell growth reached 80–90% confluence [35].

### 3.8. Cytotoxicity and Antiproliferative Activity Assays

The cytotoxicity and antiproliferative activity were determined according to the methylene blue assay [44] with some modification. In the cytotoxicity test, 100 μL of a HepG_2_ cell suspension was seeded at a density of 2.5 × 10^5^ cells/mL on a 96-well microtiter plate in a growth medium and cultured in a cell culture chamber containing 5% CO_2_ at 37 °C for 24 h. Then the adherent cells were washed with cold PBS and incubated for 24 h under 100 μL of growth medium with different concentrations of tested samples. Immediately, the cells were stained with methylene blue at 37 °C for 1 h. Then the plate was washed with deionized water for three times to clean the dye adsorbed on the cell surface. At last, 100 μL of elution buffer was added to the 96-well plate, which was shaken at room temperature for 20 min, and placed in a Spectra Max M5e multi-function microplate reader (SpectraMas, BeckMan Couter, Pasadena, CA, USA) to determine the absorbance at 570 nm.

In the antiproliferative activity test, 100 μL of HepG_2_ cells were seeded on a 96-well microplate at a density of 2.5 × 10^5^ cells/mL, and cultured in an atmosphere containing 5% CO_2_ at 37 °C for 4 h to attach the wells. Growth media with different concentrations of the samples were then added to the cell plates. The cells were cultured in a cell incubator containing 5% CO_2_ at 37 °C for 72 h and stained to be counted as described above. Cell viability was calculated as a percentage compared to the control.

### 3.9. Cellular Antioxidant Enzymes Activities Assay

The logarithmic growth phase of the HepG_2_ cells were inoculated in 6-well plates at a density of 1 × 10^6^ cells/mL. After 24 h incubation in a cell incubator, the cells were washed with PBS and treated with different concentrations of COP2-Zn, Glu-Zn, ZnSO_4_·7H_2_O, and OP-2 (with zinc ion concentrations of 40, 80, 120 μM), respectively. The cells were then dipped with 3-times pre-chilled PBS and lysed with RIPA lysate for 30 min on ice. The cells were collected and diluted with 2 volumes of PBS and centrifuged (Allegra X-15R, Beckman Coulter, Pasadena, CA, USA) at 12,000 rpm for 10 min to obtain a supernatant. Finally, the activities of the SOD, GSH-Px, and CAT of the lysed HepG_2_ cells were further determined according to the corresponding assay kit method with a microplate reader (Filter Max F5, Molecular-Devices, San Jose, CA, USA). At the same time, the contents of the proteins in the cells were measured.

### 3.10. Fluorescence and Flow Cytometry Assays

To identify the cell cycle phase distribution of HepG_2_, the cells were treated with the peptide–zinc complex COP2-Zn at concentrations of 40, 80, and 120 μM for 24 h, respectively. Then, the cells were washed, collected, and stained with propidium Iodide for 30 min in the dark. The cell cycle of HepG_2_ was determined by using a Cytomics FC 500 system with the software of Multicycle 3.0 (Beckman Center, Brea, CA, USA).

An Annexin v-fluorescein isothiocyanate apoptosis detection kit (Biyuntian Biotechnology Co., Ltd., Nanjing, China) was used to detect the apoptosis of the HepG_2_ cells. After treatment with COP-2 at concentrations of 40, 80, and 120 μM for 24 h, the cells were collected and stained with an Annexin V-FITC/PI Apoptosis Detection Kit (Biyuntian Biotechnology Co., Ltd., Nanjing, China) according to the manufacturer’s protocol. The apoptotic HepG_2_ cells were then detected by flow cytometry (Beckman Coulter, Brea, CA, USA), and the percentages of apoptotic and necrotic cells were calculated.

### 3.11. Statistical Analysis

All measurements were performed three times, and the data were expressed as the mean ± standard deviation. The statistical analysis was performed using SPSS software 19.0 (SPSS Inc., Chicago, IL, USA). The results were subjected to ANOVA and Tukey’s multiple comparison test which was used to determine the differences between the mean values; *p* < 0.05 was considered significant.

## 4. Conclusions

To prevent the toxicity of zinc ions, the zinc–peptide complex was developed to enhance the absorption of zinc ions. Oyster is rich in amino acids and high in protein, making it an ideal raw material for the production of peptide–zinc complexes. The univariate results showed that the peptide–zinc complex prepared by ultrasonic pretreatment and programmed cooling could significantly improve the chelation rate. The optimal chelating process parameters obtained through response surface optimization design are: a peptide zinc ratio of 15, pH of 6.53, and reaction time of 80 min. The chelating rate was determined to be 74.13 ± 0.62%, without a significant difference compared to the predicted value. Through ultraviolet and infrared absorption analysis, it was found that the maximum absorption peak after chelation turned red. At 1400 cm^−1^, the tensile vibration of the carboxyl group turned blue and disappeared in situ, which further indicated the production of a zinc chelate of oyster polypeptide. The results of the cell cycle and apoptosis indicated that COP2-Zn, ZnSO_4_, and OP-2 could induce S-phase cell cycle arrest, directly inhibit cell proliferation, and cause apoptosis, which is related to the increase in the antioxidant enzyme activity.

## Figures and Tables

**Table 1:** Deign and experimental results of zinc chelating oyster peptide.

Test Number	Level			Chelating Rate
	Peptide/Zinc	pH	Time (min)	(%)
1	10	6	60	72.61
2	15	6	40	63.08
3	15	5	60	65.92
4	5	6	40	61.81
5	5	6	80	60.89
6	15	7	60	70.83
7	10	6	60	70.94
8	10	7	40	66.99
9	10	7	80	69.33
10	10	5	40	63.61
11	15	6	80	76.08
12	5	7	60	64.42
13	10	6	60	69.54
14	5	5	60	59.36
15	10	6	60	71.94
16	10	6	60	69.61
17	10	5	80	63.94

**Table 2:** Analysis of variance for response surface quadratic model.

Source	df	Mean Sequence	F Value	*p* Value	Significant
Model	9	37.0597	12.7800	<0.0001	***
A	1	108.3538	37.3657	0.0005	**
B	1	43.8834	15.1331	0.0060	**
C	1	27.2362	9.3924	0.0182	*
AB	1	0.0051	0.0018	0.9677	-
AC	1	48.4186	16.6971	0.0047	**
BC	1	1.0111	0.3487	0.5734	-
A^2^	1	41.8140	14.4195	0.0067	*
B^2^	1	29.5051	10.1748	0.0153	*
C^2^	1	22.5216	7.7666	0.0270	*
Residual	7	2.8998	-	-	-
Lack of fit	3	4.2574	2.2626	0.2233	-

* represents the significant difference. (*: *p* < 0.05, **: *p* < 0.01, ***: *p* < 0.001).

**Table 3:** Amino acid compositions of a zinc-chelating oyster peptide.

Amino Acid Composition	Content (g/100 g)	Amino Acid Composition	Content (g/100 g)
Asp	2.708	Ile *	0.819
Thr *	1.040	Leu *	0.908
Ser	0.937	Tyr	0.489
Glu	2.496	Phe *	0.464
Gly	1.699	Lys *	1.260
Ala	0.704	His	0.781
Cys	0.195	Arg	1.085
Val *	0.861	Pro	1.539
Met *	0.576	Trp *	-

* represents an essential amino acid.

## Data Availability

Data are available in a publicly accessible repository.
